# Enhancement of lipase activity in non-aqueous media upon immobilization on multi-walled carbon nanotubes

**DOI:** 10.1186/1752-153X-1-30

**Published:** 2007-11-29

**Authors:** Shweta Shah, Kusum Solanki, Munishwar N Gupta

**Affiliations:** 1 Department of Chemistry, Indian Institute of Technology Delhi, Hauz Khas, New Delhi, 110016, India

## Abstract

**Background:**

Immobilization of biologically active proteins on nanosized surfaces is a key process in bionanofabrication. Carbon nanotubes with their high surface areas, as well as useful electronic, thermal and mechanical properties, constitute important building blocks in the fabrication of novel functional materials.

**Results:**

Lipases from *Candida rugosa *(CRL) were found to be adsorbed on the multiwalled carbon nanotubes with very high retention of their biological activity (97%). The immobilized biocatalyst showed 2.2- and 14-fold increases in the initial rates of transesterification activity in nearly anhydrous hexane and water immiscible ionic liquid [Bmim] [PF6] respectively, as compared to the lyophilized powdered enzyme. It is presumed that the interaction with the hydrophobic surface of the nanotubes resulted in conformational changes leading to the 'open lid' structure of CRL. The immobilized enzyme was found to give 64% conversion over 24 h (as opposed to 14% with free enzyme) in the formation of butylbutyrate in nearly anhydrous hexane. Similarly, with ionic liquid [Bmim] [PF6], the immobilized enzyme allowed 71% conversion as compared to 16% with the free enzyme. The immobilized lipase also showed high enantioselectivity as determined by kinetic resolution of (±) 1-phenylethanol in [Bmim] [PF6]. While free CRL gave only 5% conversion after 36 h, the immobilized enzyme resulted in 37% conversion with > 99% enantiomeric excess. TEM studies on the immobilized biocatalyst showed that the enzyme is attached to the multiwalled nanotubes.

**Conclusion:**

Successful immobilization of enzymes on nanosized carriers could pave the way for reduced reactor volumes required for biotransformations, as well as having a use in the construction of miniaturized biosensensor devices.

## Background

There has been considerable interest in the use of nanomaterials in biochemical applications [1-4], in particular the immobilization of proteins on nanomaterials [3-8], with nanotubes having lately attracted considerable attention [3,5-8]. Both single-walled carbon nanotubes (SWNTs) and multi-walled carbon nanotubes (MWNTs) have been used as matrices.

Immobilization is carried out using a variety of approaches [9,10], including adsorption [5,7] and covalent immobilization [6,8], techniques on which various studies have been carried out. A key parameter in any immobilization is the retention of the protein's biological activity. In this respect adsorption is assumed to be superior to other approaches, which generally utilize harsher conditions [9,10]. In most cases reported hitherto [5-8], the retention of enzymic activity upon immobilization on nanotubes, has not been high, with reports describing enzymes retaining 1–20% of their biological activity [7,8]. This is similar to the early work on macroscopic matrices and it is expected that better results would be obtained once better experience/strategies with respect to nanotubes (as matrices) were in place. It is noteworthy, however, that two recent studies report retention of biological activities over the range 55–100% [11,12].

There do not, however, appear to be any reports on the evaluation of enzymes immobilized on carbon nanotubes in non-aqueous media, which, both in the contexts of biocatalysis and bioanalysis, offer great potential [13-17].

This study reports the preparation and characterization of a high activity *Candida rugosa *lipase (CRL) immobilized on multi-walled carbon nanotubes by adsorption. Numerous applications have been found for lipases in non-aqueous media [13-17], with the *Candida rugosa lipase *one of the most frequently used in biotechnology [18]. Yet there is another reason for which a lipase was chosen for this study. Most lipases have a 'lid', which opens up upon interaction with hydrophobic surfaces, enabling them to assume an 'active conformation'[18]. Given that the surface of nanotubes is hydrophobic [3], the choice of a lipase was considered worthwhile in developing a nanobiocatalyst with high activity.

## Results and Discussion

### Adsorption of lipases on MWNT

It is well known that the retention of biological activity of an enzyme upon immobilization on a surface (immobilization efficiency) is dependent upon the number of enzyme molecules loaded onto a given surface area [9]. Table 1 shows the variation in immobilization efficiency with different quantities of enzyme, to which the same amount of nanotubes was exposed, with the highest immobilization efficiency obtained being 0.53. It has been reported that in the case of some lipases, the presence of an inert protein, such as albumins, gave higher immobilization efficiencies with hydrophobic surfaces like accurel™ [19].

**Table 1:** Immobilization of *Candida rugosa *lipase on MWNT. Different units of CRL were immobilized on 1 mg of MWNTs. The hydrolytic activity was determined on MWNTs, supernatant and washings. All experiments were carried out in duplicate and the results within each pair differed by < 5%.

**Loaded Units**	**Supernatant + washings**	**Expressed activity (B)**	**Theoretical activity (A)**	**B/A**
0.74	0.04	0.30	0.70	0.42
1.48	0.24	0.65	1.24	0.52
2.9	0.29	1.39	2.60	0.53
5.8	1.63	2.04	4.17	0.48

The commercial preparation of CRL that has been used contains 13 μg protein/mg solid. Given the high surface area of nanotubes, 2.9 units (entry 3 Table 1, corresponding to immobilization efficiency of 0.53) constitute a very low protein load region. As pointed out by Bosley and Pielow [19], in such situations the enzyme attempts to maximize its contact with the surface, leading to undesirable conformational changes and hence loss of activity. This is overcome by the addition of other proteins that block the 'high affinity' sites on the support, or simply reduce the surface area available to the enzyme. Table 2 shows that the presence of bovine serum albumin did influence immobilization efficiency in a positive manner. With an optimum amount of BSA, an immobilization efficiency of 0.97 could be obtained. This implies that almost all the enzyme molecules immobilized on the MWNT retained their full hydrolytic activity.

**Table 2:** Immobilization of *Candida rugosa *lipase on MWNTs in presence of BSA. CRL (3 units) were immobilized on 1 mg of MWNTs in presence of varying amount of BSA. The hydrolytic activity was determined on MWNTs, supernatant and washings. All experiments were carried out in duplicate and the results within each pair differed by < 5%.

**BSA amount**	**Supernatant + washings**	**Expressed activity (B)**	**Theoretical activity (A)**	**B/A**
0	0.29	1.39	2.60	0.53
0.25	0.61	1.55	2.39	0.64
0.5	1.07	1.52	1.93	0.78
1	1.53	1.35	1.47	0.88
2	1.66	1.31	1.34	0.97
3	2.22	0.54	0.78	0.69

### Catalytic performance in non-aqueous media

While many applications have been found for immobilized lipase, such as fat splitting *etc *in the conventional aqueous medium, the last two or three decades have witnessed many interesting applications in nearly-anhydrous organic solvents [13-17]. These applications are based upon esterification/transesterification reactions, which become possible in such media owing to the absence of bulk water. Table 3 gives the initial rates of transesterification in dry hexane. The increased initial rates (in non-aqueous media) with immobilized forms of an enzyme are believed to be due to decreased mass transfer limitations. Enzyme powders in organic media are believed to form physical aggregates that diminish the substrate's access to the enzyme active site [9,13,15-17]. The immobilization leads to 'spreading' enzymes on the surface. In addition to this, the immobilization itself also leads to stabilization of the native enzyme structure. In the case of CRL, it is likely that a third factor plays an important role. Most of the lipases, including CRL, have a unique structural feature – a lid or flap consisting of an amphiphilic alpha-helix peptide covering the active site. The presence of an interphase (example water-organic interphase) moves the lid and exposes the active site [20]. Hydrophobic supports are also known to cause similar molecular changes [18,21]. Such 'open lid' conformations are known to be more active.

**Table 3:** Initial transesterification rates exhibited by *Candida rugosa *lipase immobilized MWNTs in different reaction media. All experiments were carried out in duplicate and the results within each pair differed by < 5%.

**Lipase preparations**	**Reaction medium**	**Initial rates (μmoles min^-1 ^h^-1^)**	**Time increase**
pH tuned		0.24	1
Immobilized on MWNTs (In presence of BSA)	Hexane	0.36	1.5
			
Immobilized on MWNTs		0.54	2.25

pH tuned		0.12	1
Immobilized on MWNTs (In presence of BSA)	[Bmim] [PF_6_]	1.08	9
			
Immobilized on MWNTs		1.62	14

Interactions with the hydrophobic surfaces of nanotubes are expected to result in the 'open lid', as well as more active lipase conformations. Another observation was that in such media, the lipase immobilized in the absence of BSA gave somewhat higher initial rates. This may be explained by the fact that enzymes in nearly anhydrous media are extremely rigid molecules. In fact, it has been reported that some enhancement of flexibility either through urea denaturation [22] or three-phase partitioning [23] results in higher enzymic biological activity. It is not unlikely that the conformational changes leading to limited denaturation as a result of interactions with the carbon nanotube had acquired the necessary conformational flexibility. The interactions of the enzyme with the carbon nanotube in the presence of BSA, on the other hand, had a very close to native, and hence normal, rigid structure.

Lately, ionic liquids have also emerged as an alternative reaction medium for enzyme catalysis [24,25]. Described as 'green solvents' and 'designer solvents', these room temperature ionic liquids release practically no volatile organic compounds into the atmosphere [24-26]. The water immiscible ionic liquids have generally been reported as being better reaction media than water miscible ionic liquids for enzyme catalysis [24,25], with [Bmim] [PF6] being one of the most frequently used water immiscible ionic liquids for enzyme catalysis [24,25,27,28].

The same reaction, that is, formation of butylbutyrate, was also carried out in [Bmim] [PF6]. The lipase immobilized on MWNT (in the absence of BSA) showed a 14-fold increase in the initial rates of transesterification over those shown by pH tuned lyophilized powders (Table 3).

### Biotransformations with the lipase immobilized on MWNT

**Figure 1:**
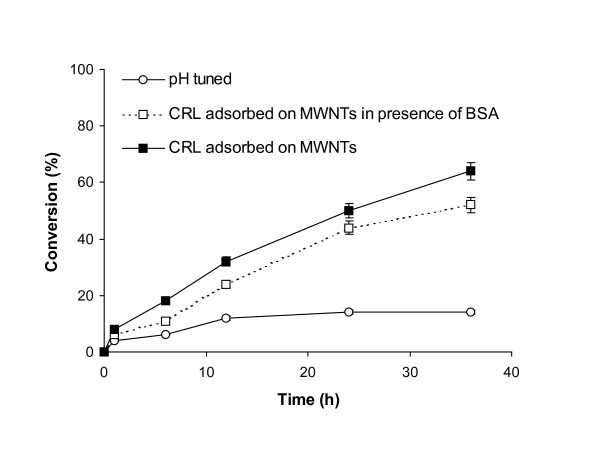
Transesterification of ethyl butyrate with butanol in hexane. Each point represents the outcome of a pair of readings.

**Figure 2:**
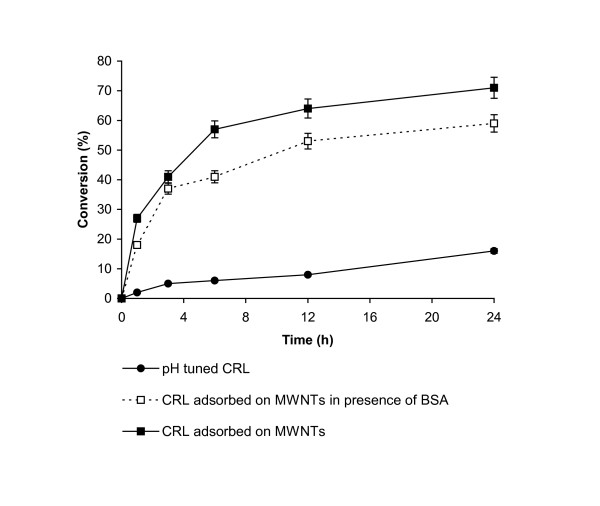
Transesterification of ethyl butyrate with butanol in [Bmim] [PF6]. Each point represents the outcome of a pair of readings.

As the lipase immobilized on MWNT showed higher initial transesterification rates in hexane, its utility in the biotransformation was evaluated by the conversion of ethyl butyrate to butyl butyrate over larger reaction periods. Figure 1 shows that CRL immobilized on MWNT gave much better conversion rates. The immobilized enzyme gave 64% conversion after 24 h, whereas the free enzyme gave 14% conversion after the same reaction period. Even in [Bmim] [PF6] the immobilized enzyme was a far better catalyst (Figure 2). The immobilized enzyme showed 71% conversion, whilst the free enzyme gave 16% conversion after 24 h. Again, the enzyme immobilized in the absence of BSA performed better. In a control where only MWNTs without enzyme were added, no transesterification activity could be obtained either in hexane or in the ionic liquid.

### Enantioselectivity of lipase immobilized on MWNT

The kinetic resolution of racemates to obtain chiral compounds has emerged as a major application of enzymes [15-19,29]. Table 4 shows that free enzyme showed poor transesterification rates during kinetic resolution of (±) 1-phenyl ethanol. However, the enzyme immobilized on MWNT showed significant improvement in percentage conversion with a very high enantiomeric excess of > 99. An enatioselectivity of 360 (with a 36 h reaction time) was calculated according to Chen's equation [30] (note: in the kinetic resolution, 50% would be the maximum). No transesterification could be obtained when a control was run in which only MWNTs were added without lipase.

**Table 4:** Transesterification of (±) 1-phenyl ethanol in ionic liquid with *Candida rugosa *lipase immobilized on MWNTs. All experiments were carried out in duplicate and the results within each pair differed by < 5%.

**Entry**	**Lipase preparation**	**Time (h)**	**Conversion (%)**	**ee (%)**	**E^a^**
1	Free CRL	12	1	-	-
2	Free CRL	24	2	-	-
3	Free CRL	36	5	-	-
4	CRL adsorbed on MWNTs	12	17	>99	250
5	CRL adsorbed on MWNTs	24	34	>99	350
6	CRL adsorbed on MWNTs	36	37	>99	360

^a^Enantioselectivity (E) = ln [1 - c(1 + eep)]/ln [1 - c(1 - eep)], where c = ees/(ees + eep).

### TEM of CRL immobilized on MWNT

**Figure 3:**
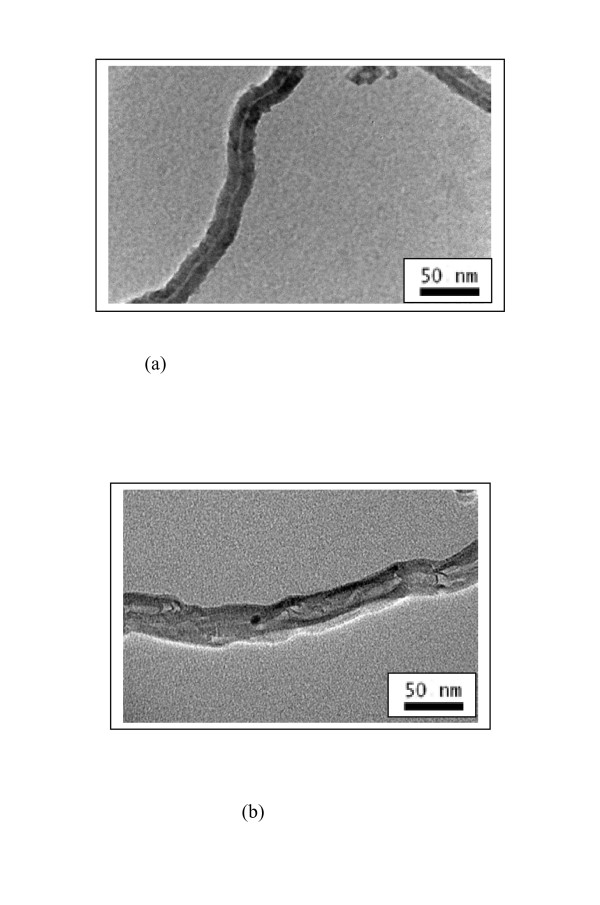
(a) TEM images of MWNTs; (b) CRL absorbed on MWNTs.

TEM images (Figure 3) showed that the enzyme was actually present on the MWNT. In many cases of immobilized enzymes being used in organic media (immobilization on celite is a well-known example), it is believed that an immobilization matrix like celite simply acts as a dispersing agent reducing mass transfer constraints [31]. Thus such systems do not constitute true immobilized enzymes. In the present instance, TEM showed that the system could be described as CRL immobilized on MWNT. Comparing the diameters of the nanotubes with (20 ± 5 nm) and without the enzyme (30 ± 5 nm) (the diameter values represent the average of 10 TEM images in each case), it appears that lipase was physically present on the nanotubes. The enzyme seemed to cover the entire surface of the nanotube, reflecting the evenness of the enzyme 'coating'. Given the propensity of the lipase to form aggregates on surfaces [32], it was thought very unlikely that only a monolayer of lipase molecules had formed on the nanotubes.

## Conclusion

There is increasing interest in the miniaturization of technological devices, with studies on the immobilization of enzymes on nanotubes, part of this trend. The present work illustrates that lipase-nanotube systems have some unique features, for instance immobilization on nanotubes actually led to considerable enhancement of enzymic activity in non-aqueous media. In this study, we have reported for the time the performance of an enzyme on carbon nanotubes in non-aqueous media.

### Experimental

CRL was a gift from Amano Enzyme Inc., Nagoya, Japan. Multi-walled carbonnanotubes (OD = 10–30 nm, ID = 3–10 nm, length = 1–10 μm, purity 90 + %, Cat. No. 636517) and p-Nitrophenylpalmitate (p-NPP) were obtained from Sigma Chemical Co., St. Louis, USA. The ionic liquid was from Acros Organics, USA. The purity of [Bmim] [PF6] as specified by the vendor was 99.6% (by HPLC) with water content of 0.05% v/v by Karl-Fisher. All solvents were of low water grade (< 0.005% water (vv^-1^)) and obtained from J. T. Baker, USA. These solvents were further dried by gentle shaking with 3 Å molecular sieves (E. Merck, Mumbai, India).

### Adsorbtion of Lipase on MWNTs

The MWNTs (1 mg) were dispersed by sonication (Elma transsonic digital ultrasonic unit, model T 490 DH, Germany) at a fixed frequency of 40 kHz and at 110 W power rating) in 0.5 ml of 50 mM phosphate buffer pH 7.0 for 30 min, followed by addition of 0.5 ml of the lipase solution (containing varying amount of the lipase). The mixture was kept at 20°C with constant shaking at 200 rpm for 3 h. After, incubation, mixture was centrifuged at 8,000 *g *for 10 min at 20°C. The supernatant was removed and MWNTs were washed with 1 ml of 50 mM phosphate buffer at pH 7.0 until no hydrolytic activity was detected in the washings.

### Transesterification reaction

Ethyl butyrate (60 mM) and *n*-butanol (120 mM) were added to a vial containing 1 ml of hexane/ionic liquid followed by addition of free lipase/lipase adsorbed (10 mg) on MWNTs. The reaction mixture was incubated at 35°C at 200 rpm. The aliquots were withdrawn at different time periods and analyzed by GC. A control was run in which MWNTs were added without lipase.

### Protein estimation

The protein concentration was determined according to Bradford's method, using bovine serum albumin as the standard protein [33].

### Lipase assay

The hydrolytic activity of lipase was monitored by following the rate of hydrolysis of p-nitrophenylpalmitate spectrophotometrically at 410 nm [34].

### GC analysis

The alkylesters were analyzed on Agilent Technologies 6890 N network GC systems, USA, with a flame ionization detector. The capillary specifications were: a column length of 30 m, internal diameter of 0.25 mm. A nitrogen carrier gas at a constant pressure of 4 Kg/cm^2 ^was used. The column oven temperature was programmed over the range of 150 to 250°C at 10°C min^-1 ^with injector and detector temperatures at 240 and 250°C, respectively.

### Kinetic resolution of (±)-1-phenylethanol

The alcoholysis between (±)-1-phenylethanol (1 mmol) and vinyl acetate (1 mmol) was carried out in 1 ml ionic liquid followed by addition of CRL/CRL immobilized on MWNTs at 30°C with constant shaking at 250 rpm. The samples were taken out at different time intervals and analyzed by HPLC. A control was run in which MWNTs were added without lipase.

### HPLC analysis

For HPLC analysis, 50 μl of the reaction mixture was extracted with 500 μl n-hexane-propan-2-ol (97.5:2.5) mixture. The extract was analyzed by HPLC using Chiracel OD12 RH column (Diacel, Japan). The eluent consisted of 96.5% (vv^-1^) n-hexane, 3% (vv^-1^) propan-2-ol and 0.5% (vv^-1^) ethanol with a flow rate of 1 ml min^-1 ^and detection was carried out with a UV detector at 254 nm.

### Transmission electron microscopy (TEM)

Transmission electron micrographs were recorded on a FEI Philips Tecnai F20 (200 kV, FEG) instrument. A drop of MWNTs/lipase adsorbed on MWNTs dispersed in distilled water was placed on a copper grid and dried.

## Authors' contributions

SS performed almost all the experiments and drafted sections on materials and methods and drew figures. KS carried out immobilization for recording TEM and their analysis. She was also involved with drafting the entire manuscript at the revision stage. MNG participated in the design of experiments, discussions, interpretation of results and writing of most of the manuscript. All authors approved the final manuscript.
